# Pulmonary Large Cell Neuroendocrine Carcinoma: A Rare Type of Non-Small Cell Lung Cancer

**DOI:** 10.7759/cureus.14734

**Published:** 2021-04-28

**Authors:** Thomas G Ng, Hyo-bin Um, Mark Forsberg, Usha Trivedi, Jason George

**Affiliations:** 1 Internal Medicine, Rutgers University, Newark, USA; 2 Radiology, Rutgers University, Newark, USA; 3 Pulmonary and Critical Care, East Orange Veterans Affairs, East Orange, USA

**Keywords:** large cell lung cancer, large cell neuroendocrine carcinoma, rutgers njms, metastatic non-small cell lung cancer, veteran affairs

## Abstract

Pulmonary large cell neuroendocrine carcinoma (LCNEC) is an uncommon type of non-small cell lung cancer (NSCLC) with an incidence of approximately 3% of all lung cancer diagnoses. The patient was a 60-year-old male with a 90-pack year smoking history who presented with dyspnea on exertion and productive cough for five weeks. Decreased breath sounds without respiratory distress and generalized cachexia were noted on the initial physical exam. Laboratory results were unremarkable except for chronic microcytic anemia. Computed tomography revealed extensive lymphadenopathy of the paratracheal, paraaortic, hilar, and nodes surrounding the left pulmonary arteries. Additionally, there were areas of necrosis in the left upper lobe, lingula, and left lower lobe with extensive pleural thickening extending to the abdomen and subcutaneous tissue of the anterior chest wall. Biopsy and staining showed disorganized tight cell clusters with irregular and prominent nuclei and numerous lymphocytes consistent with LCNEC. Immunohistochemistry was positive for neural cell adhesion molecule CD56 and synaptophysin, which was indicative of neuroendocrine origin. It was also positive for pan-cytokeratin antibody AE1 and AE3 and cytokeratin (CAM) 5.2, which arise from epithelial origin consistent with NSCLCs. Lastly, the patient’s tissue was positive for thyroid transcription factor-1, which confirmed the tumor’s primary lung origin. This combination of neuroendocrine and primary lung tumor markers, in conjunction with the histology, confirmed the patient’s diagnosis of LCNEC.

## Introduction

Pulmonary large cell neuroendocrine carcinoma (LCNEC) was first classified by the World Health Organization as a variant of large cell carcinomas under the broad category of non-small cell lung cancer (NSCLC). Like other lung cancers, risk factors include age, male sex, and exposure to tobacco smoke. Most NSCLCs are lung adenocarcinomas and lung squamous cell carcinomas. LCNEC represents less than 3% of all lung cancer diagnoses [1]. 

In the lung, the bronchioles are defined by major cell types of ciliated, columnar, undifferentiated, secretory neuroepithelial bodies, and basal cells. The alveoli are composed of type 1 and type 2 cells, as well as sporadic neuroendocrine (NE) cells. Each of these cell lines can develop into oncogenic progenitors leading to various types of pulmonary tumors. The molecular characterization of major lung tumor subtypes and their origins has led to both academic and clinical distinctions. Lung squamous cell carcinoma arises from basal cells, hence their keratin expression and histology. Small cell lung cancer (SCLC) arises primarily from the NE cells. LCNEC has been hypothesized to arise from these same NE cells although there is conflicting evidence regarding the exact epithelial cell types. These tumors develop from distinct transcriptional subgroups and genetic drivers with potentially significant therapeutic implications. It is this neuroendocrine origin, combined with aggressive mutations, which leads to a high-grade, poorly differentiated carcinoid state.

Due to its neuroendocrine origin, LCNEC are often compared to SCLC, as there is some data to suggest molecular and therapeutic response overlap in these poorly understood recalcitrant tumors. However, there are also crucial differences in cytomorphological features and clinical presentation. Unlike SCLC, LCNEC arises peripherally more than centrally, and this variable presentation contributes to a greater average stage at the time of detection [2]. The diagnosis of LCNEC requires histology that demonstrates a high-grade carcinoma with neuroendocrine characteristics and tumor markers to confirm lung and neuronal origin. Here, we present a case of a United States serviceman who was admitted to our institution and was found to have LCNEC.

## Case presentation

**Figure 1:**
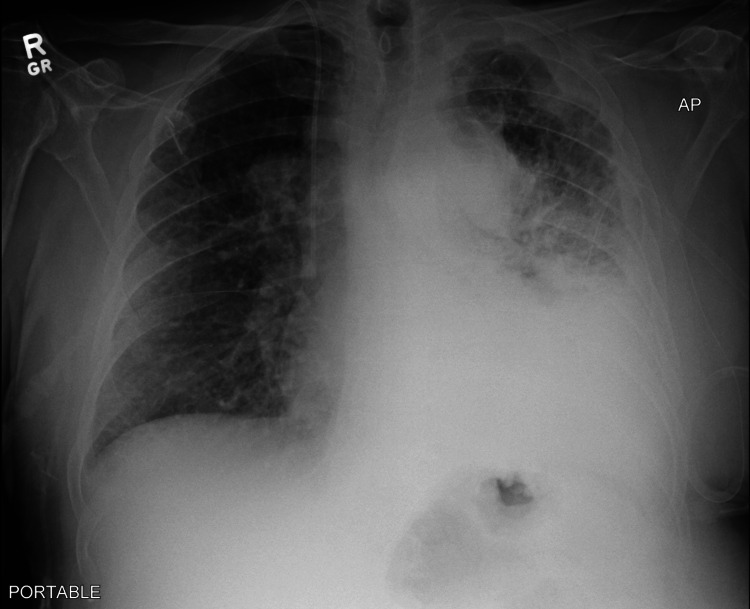
Initial chest radiograph showing large, left-sided opacity consistent with a moderate, left-sided pleural effusion and an underlying infiltrate with atelectasis There were also increased densities in the left upper lobe as well. In addition, extensive mediastinal lymphadenopathy was noted, as demonstrated by the right paratracheal widening.

**Figure 2:**
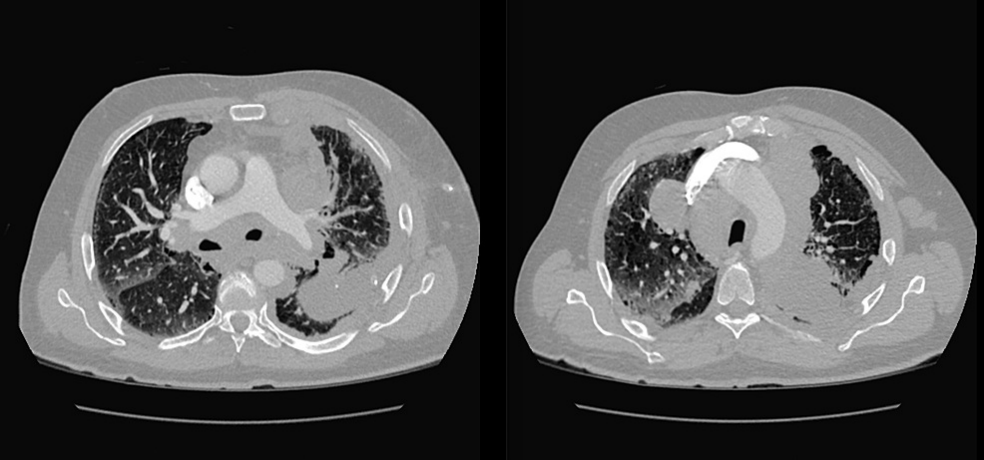
Large lobular masses in the anterior and posterior mediastinum, pretracheal, and para-aortic regions, which represent a conglomerate of lymph nodes This conglomerate encases and compromises the segmental branches of the bronchus and pulmonary arteries bilaterally and was seen extending to the left upper lobe lingula and left lower lobe segments.

**Figure 3:**
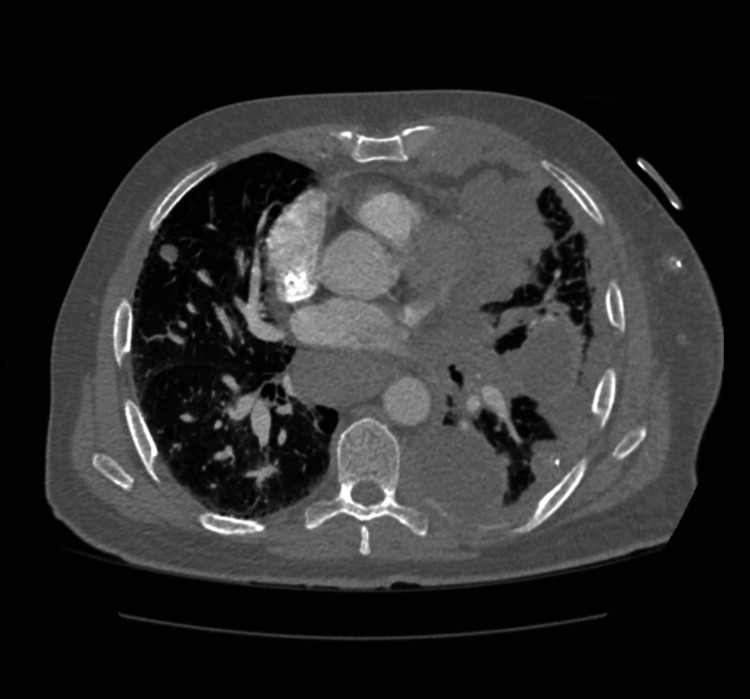
Necrosis encompassed the left upper lobe, lingula, and left lower lobes There was also extensive pleural thickening and inflammatory changes extending from the left lung that involved the abdomen and the subcutaneous tissue of the anterior chest wall.

The patient was a 60-year-old male with a 90-pack/year smoking history and exposure to Agent Orange during his service, who presented with 30-pound occult weight loss, worsening dyspnea on exertion, and a productive cough over five weeks. One year prior, he was found to have a centrally localized lesion on low-dose computed tomography (CT) screening but did not follow up for further evaluation. On admission, he was found to be tachypneic and tachycardic on examination with decreased breath sounds on the left side and ipsilateral axillary lymphadenopathy. Laboratory findings were significant for chronic microcytic anemia with a hemoglobin of 8.9 g/dL without significant elevations in inflammatory markers. An initial chest radiograph showed mediastinal widening due to lymphadenopathy with moderate left-sided pleural effusion and underlying infiltrate and atelectasis (Figure 1). A subsequent contrast-enhanced CT revealed extensive lymphadenopathy of the paratracheal, para-aortic, and hilar lymph nodes. There was also a collection of lymph nodes encasing the bronchus and pulmonary arteries leading to the left upper lobe, lingula, and left lower lobe, with some involvement of the right side suspicious for lymphoma (Figure 2). Additionally, there were lobular areas consistent with necrosis in the left upper lobe, lingula, and left lower lobe, as well as the parenchymal lymph nodes. Extensive pleural thickening extending to the abdomen and subcutaneous tissue of the anterior chest wall was also identified (Figure 3).

Core biopsy of the mediastinum revealed disorganized tight cell clusters with irregular and prominent nuclei and numerous lymphocytes consistent with NSCLC. Immunohistochemistry confirmed a neuroendocrine origin with positive neural cell adhesion molecule CD56, synaptophysin, and thyroid transcription factor-1 (TTF-1) markers consistent with LCNEC. Due to the extensive disease burden, surgery was deferred, and the patient was started on carboplatin and etoposide with the addition of atezolizumab a few weeks later. However, the patient was unable to tolerate his treatment regimen given the extent of the disease. He later developed pneumonia, continued to decline despite broad-spectrum antibiotics and aggressive treatment, and passed away due to complications of septic shock approximately two months after the initial presentation.

## Discussion

Lung adenocarcinomas and squamous cell carcinomas are the most common types of NSCLC. LCNEC, a rare type of NSCLC, is often not considered in the differential and misdiagnosed. This patient, who had a significant smoking and occupational exposure history, presented with nonspecific symptoms typical of most lung cancers. In approximately 64% of recorded cases, LCNEC arises peripherally instead of centrally near the bronchi [3]. This patient’s lung disease progression was atypical in that the initial imaging showed that the cancer originated centrally instead of peripherally. These tumors are bulky with cavitation, inflammation, and necrosis, likely due to the high mitotic rate and angioinvasion, more typical of the poorly differentiated and aggressive LCNEC. Histologically, LCNEC cells are often described as high-grade, large, polygonal, pleomorphic cells with neuroendocrine morphology with high mitotic rates surrounded by large areas of necrosis and they are often larger than the surrounding lymphocytes with a small nuclear-to-cytoplasmic ratio, typical of large cell lung cancers [4]. In addition to histology, the confirmation of LCNEC requires at least one positive neuroendocrine immunohistochemical specific marker such as chromogranin A, synaptophysin, or CD56, and as well as TTF-1 or mucin to indicate a primary lung origin [5].

Immunohistochemistry of the patient’s lung tissue was positive for CD56 and synaptophysin indicative of neuroendocrine origin. CD56 is a marker for neural cell adhesion molecules used to activate fibroblast growth factor receptors. Synaptophysin is only found on neural or neuroendocrine tissues. The tissue was also positive for pan-cytokeratin antibody AE1 and AE3 and cytokeratin 5.2 (CAM 5.2), which arise from epithelial cells, the proposed progenitor of LCNEC. Moreover, LCNEC is also often associated with elevated expression of cytokeratin-7 (CK7), cytokeratin-8 (CK18), E-cadherin, and beta-catenin, however, these specific tumor markers were not expressed in this case [6]. Lastly, the tissues were positive for TTF-1, a substance produced by alveolar type 2 cells, which confirmed cancer’s primary lung origin. Of note, pulmonary adenocarcinomas can also exhibit TTF-1, but such a tumor is histologically distinct from LCNEC. This combination of neuroendocrine and primary lung tumor markers, in conjunction with the histology, confirmed the patient’s diagnosis (Table 1). Aggressive follow-up for this patient could possibly have been useful in prompting earlier treatment, however, given the patient's disease and multiorgan involvement with initial presentation, there was still a high likelihood of significant morbidity and mortality. While guidelines specific for LCNEC treatment are still being developed, therapies parallel those for other SCLC. However, there is increasing evidence of molecular and clinicopathological heterogeneity between the two diseases with potential therapeutic consequences. Based on tumor size and involvement, surgical resection can be performed as part of initial treatment, but chemotherapy with a platinum-based regimen is often initiated in cases with severe tumor involvement. Recent data from the incorporation of immunotherapy alongside chemotherapy regimens may provide future avenues for treatment protocols and new therapeutic targets [7].

**Table 1:** Diagnostic criteria for pulmonary large cell neuroendocrine carcinoma High Power Film (HPF), CD56 (neural cell adhesion molecule CD56), Cytokeratin 5.2 (CAM 5.2), Thyroid Transcription Factor-1 (TTF-1)

Table 1. Diagnostic Criteria for LCNEC		
Origin	Peripheral > Central	
Histology	Large, polygonal cells with abundant cytoplasm	
	Low nuclear to cytoplasm ratio (N/C)	
	Nuclear pleomorphism with visible and prominent nuclei	
Patterns	Trabecular, palisading, rosette formation	
Mitotic Rate	High (>10 mitoses/10 HPF)	
Markers	Neuroendocrine	CD56, synaptophysin, chromogranin
	Epithelial	Pan-cytokeratin antibodies (AE1/AE3), CAM 5.2
	Alveolar	Thyroid Transcription Factor-1 (TTF-1)

## Conclusions

LCNEC is a rare subtype of NSCLC associated with poor prognosis and a five-year survival rate of 15%-57% approaching 0% for stage IV disease. Due to its low incidence, LCNEC is often an overlooked diagnosis requiring both histologic and immunohistochemical confirmation. Surgical resection is usually recommended for localized tumors. If the tumor is deemed inoperable, platinum-based chemotherapy based on SCLC treatment protocols are utilized since both tumors are of neuroendocrine origin. However, there is conflicting evidence as to whether platinum/etoposide therapy is as effective in treating LCNEC as it is for SCLC. More research is needed to better evaluate and improve the management of this recalcitrant cancer. Our case demonstrates that, although uncommon, LCNEC should be considered as a differential when evaluating a patient with extensive lung pathology. It also contributes much-needed data to the development of effective treatment protocols for this devastating and poorly understood disease.
